# The influence of physiotherapy intervention on patients with multiple sclerosis–related spasticity treated with nabiximols (THC:CBD oromucosal spray)

**DOI:** 10.1371/journal.pone.0219670

**Published:** 2019-07-30

**Authors:** Alessandro Enrico Grimaldi, Laura De Giglio, Shalom Haggiag, Assunta Bianco, Antonio Cortese, Sebastiano Giuseppe Crisafulli, Fabrizia Monteleone, Gerola Marfia, Luca Prosperini, Simonetta Galgani, Massimiliano Mirabella, Diego Centonze, Carlo Pozzilli, Letizia Castelli

**Affiliations:** 1 MS Center Sant’Andrea Hospital, Sapienza University of Rome, Rome, Italy; 2 Department of Human Neuroscience, Sapienza University of Rome, Rome, Italy; 3 Multiple Sclerosis Clinical Centre, San Camillo-Forlanini Hospital, Rome, Italy; 4 Multiple Sclerosis Unit, Fondazione Policlinico Universitario Agostino Gemelli IRCCS, Università Cattolica, Rome, Italy; 5 MS Center, Department of Systems Medicine, Tor Vergata University, Rome, Italy; 6 Unit of Neurology and Neurorehabilitation, IRCCS Neuromed, Pozzilli, Italy; 7 IRCCS Fondazione Don Carlo Gnocchi, Milan, Italy; Heinrich-Heine-Universitat Dusseldorf, GERMANY

## Abstract

**Background:**

Nabiximols (THC/CBD Oromucosal Spray, Sativex) is used as an add-on therapy to treat moderate to severe spasticity of Multiple Sclerosis (MS).

**Objectives:**

To examine the impact of physiotherapy (PT) programs on effectiveness and persistence of nabiximols treatment in people with MS-related spasticity.

**Methods:**

This is an observational multicenter study with a follow-up period of 12 weeks, conducted in routine care settings in Italy. Patients with moderate to severe MS-related spasticity who started nabiximols were included. Spasticity was evaluated by the patient-rated 0–10 numerical rating scale (NRS). Clinical data were collected at baseline (T0), 4 weeks (T1) and 12 weeks (T2) months after enrollment.

**Results:**

A total of 297 MS patients were selected, 290 completed the 3 months follow-up period. Mean NRS scores were 7.6 ± 1.1 at T0, 5.8 ± 1.4 at T1 and 5.5 ± 1.5 at T2. At T1, 77% of patients reached ≥20% improvement (initial response, IR); 22% reached ≥30% improvement (clinically relevant response, CRR). At T1, patients undergoing PT had a higher probability to reach CRR (Odds Ratio = 2.6 95% CI 1.3–5.6, p = 0.01). Nabiximols was discontinued in 30/290 (10.3%) patients at T1 (early discontinuers) and in 71/290 (24.5%) patients at T2 (late discontinuers). The probability of being late discontinuers was reduced in patients undergoing PT (Hazard Ratio = 0.41; 95% CI 0.23–0.69, p = 0.001).

**Conclusions:**

Our real-life study confirms nabiximols’ effectiveness in MS-related spasticity and suggests that the association of a PT program may improve overall response and persistence to nabiximols treatment.

## Introduction

Spasticity, which consists in an increase in muscular tone, is one of the most frequent symptoms associated with multiple sclerosis (MS), occurring in up to 80% of MS patients to some degree [1]. MS spasticity is associated with other MS-related symptoms, having a severe impact on patients’ quality of life [2].

Recommended first-line medications for generalized spasticity such as baclofen, tizanidine, gabapentin, and others, have demonstrated limited clinical benefit in reducing spasticity-associated symptoms [3–4]. Since the introduction of tetrahydocannabinol (THC) and cannabidiol (CBD) oromucosal spray (nabiximols, trade name Sativex), clinicians dispose of a new option for the management of generalized spasticity in MS patients resistant to first line antispastic agents [5–7].

Nabiximols contains THC and CBD in a 1:1 ratio. THC binds to cannabinoid receptors, whose activation modulates muscle tone; on the other hand, the high concentrations of CBD are supposed to reduce the psychoactive properties of THC [8]. Several real-world studies have demonstrated the efficacy of nabiximols in moderate to severe MS-related spasticity [9–13].

Physical activity and particularly exercise are important non-pharmacological tools in MS rehabilitation because they counteract many of the multifaceted consequences of the disease.

Physiotherapy (PT) interventions can be safe and beneficial option for spasticity in people with MS. PT interventions include a wide range of therapeutic approaches such as exercise training, therapeutic standing, shock waves, electrical stimulation, and vibration. The PT interventions are intended to maintain the muscle length, prevent contracture, and change mechanical properties of the musculoskeletal system and plasticity within the central nervous system. PT interventions also seem a safe treatment, with adverse events and withdrawals being minor and rare [14–15].

The main purpose of our study was to evaluate the combined effect of nabiximols and PT in the treatment of MS-related spasticity.

## Materials and methods

### Design and setting

In this multicentre observational study, we collected clinical data from 5 MS centers in Rome (Italy).

Eligible patients have started nabiximols between January 1, 2013 and October 31, 2016. All patients met the prescriptive criteria of the local regulatory authority for nabiximols as add-on treatment, as described in detail previously [11]: MS with age higher than 18 years, with spasticity (assessed with 0–10 Numerical Rating Scale (NRS) score) ≥4 (moderate to severe) and resistant to other antispastic drugs. Presence of severe cardiovascular disease, pregnancy, history of mental illness, use of cannabis and/or other psychoactive drugs represented exclusion criteria.

Consecutive patients were included in the study at the start of nabiximols treatment (baseline or T0) and followed up over 12 weeks; data were collected at baseline (T0), after 4 (T1) and 12 weeks (T2). The evolution of MS-related spasticity was evaluated by the NRS patient-reported scale (0 = no perceived spasticity, 10 = maximal perceived spasticity) [16]. We evaluated MS physical disability using the Expanded Disability Status Scale (EDSS) [17]. Patients’ attendance to PT sessions and its frequency were assessed through ad hoc interview. The primary goal of the PT program was to improve physical capabilities thus enhancing functional and daily activities. All physical activities were goal directed and personalized according to the participant's specific needs. Goal directed physical therapy (a 45 to 50 minutes session, at least once, usually three times a week including at least: assisted passive and active mobilization of upper and lower limbs; strategic stretching; stand/sit routines; coordination/deambulation training with and without rehabilitative aids) aimed at improving balance and gait, and decreasing spasticity.

Data on clinical response to nabiximols and discontinuation reasons were collected. Response to treatments was defined according to previous studies [16]: nabiximols impact was described using the initial response (IR) threshold (*Initial Responders* patients), defined as ≥20% improvement of spasticity measured with NRS score versus baseline (T0), and clinically relevant response (CRR) threshold (*Relevant Responders* patients), defined as ≥30% improvement in NRS score compared with baseline (T0). Patients who did not show improvement, or in whom it did not reach the IR threshold (<20%), were classified as *Non Responders* (NR).

Written informed consent was obtained from all patients. This study was approved by the Committee on Ethics of the Sapienza University (prot. 4253 for Registry SM001 + SM002).

### Statistical analysis

Means and standard deviations (SD) for continuous variables and number of observations and percentage for categorical variables were used. The differences between continuous variables were evaluated by the non-parametric Mann-Whitney test, while the differences between categorical variables were evaluated by the Yate’s corrected version of Chi-squared test. The following baseline variables were included in the analysis: sex, disease duration, EDSS score, disease course (RR, SP or PP), NRS score, disease-modifying therapy, ongoing PT. Predictors of response to therapy were investigated using a classification and regression tree-based analysis [18]. Such analysis allowed to identify the baseline variables associated with the 3 nabiximols response categories (IR, CRR, NR). The order of importance of these predictive variables and the specific cut-offs were automatically identified using an algorithm called exhaustive CHAID (Chi-squared Automatic Interaction Detection).

By means of a multivariate logistic regression analysis with step-wise approach, the Odds Ratios (OR) values and the respective 95% confidence intervals of the probability of CRR or IR were also calculated.

Patients were consecutively divided according to the persistence in treatment into three groups: *Continuers*, i.e. patients that were still taking therapy at the last follow-up; *early discontinuers*, i.e. patients that suspended therapy at T1 (within a month), and *late discontinuers*, i.e. patients that suspended therapy after the first 4 weeks of treatment. The reasons for nabiximols suspension in the two groups (early versus late discontinuers) were investigated according to a contingency table.

Baseline variables associated with discontinuation of nabiximols were studied in early and late discontinuers by means of a Cox multivariate regression. As main temporal variables, either time (in days) between start and discontinuation of therapy or between start of therapy and last available visit were chosen.

All p-values lower than 0.05 in either direction were considered statistically significant. Data were analyzed using the Statistical Package for Social Sciences 16.0 software (IBM SPSS, Chicago, Ill, USA).

## Results

### Patient population

Baseline demographic and clinical parameters of 297 MS patients are shown in Table 1.The study population included 121 men (41%) and 176 women (59%), aged (mean±SD) 53.4 ± 9.8 years and with a mean disease duration of 18.1±8.7 years. A diagnosis of Relapsing-remitting MS (RRMS) was found in 22% of the patients, 63% had Secondary Progressive MS (SPMS), and 15% Primary Progressive MS (PPMS). The mean EDSS score was 6.3 ± 1.3 points. One hundred-forty patients (47%) were under DMT for MS (Table 1).

**Table 1:** Baseline demographic and clinical data.

	Patients (n = 297)
Male (%)	121 (41%)
Female (%)	176 (59%)
Age (mean ± SD)	53.4 ± 9.8
Disease duration (mean ± SD)	18.1 ± 8.7
Relapsing Remitting MS (%)	66 (22%)
Secondary Progressive MS (%)	186 (63%)
Primary Progressive MS (%)	45 (15%)
Baseline EDSS (mean ± SD)	6.3 ± 1.3
Baseline NRS score (mean ± SD)	7.6 ± 1.1
Use of DMT	140 (47%)

SD = Standard Deviation. MS = Multiple Sclerosis. EDSS = Expanded Disability Status Scale. NRS = Numeric Rating Scale.

In Table 2 we report the concomitant use of other anti-spastic drugs. Concomitant treatment with other anti-spastic agents occurred in 241 (81.1%) patients, of them 143 (48.1%) were on monotherapy and 98 (33%) on polytherapy.

**Table 2:** Antispastic treatments other than nabiximols in MS patients (n = 297).

Drug	N (%)
None	56 (18.9%)
Baclofen	210 (70.7%)
Tizanidine	20 (6.7%)
Botulinum toxine	8 (2.7%)
Intrathecal Baclofen	1 (0.3%)
Benzodiazepines	14 (4.7%)
Gabapentin	69 (23.2%)
Pregabalin	38 (12.8%)
Eperisone	2 (0.7%)
Monotherapy	143 (48.1%)
Polytherapy	98 (33.0%)

### Nabiximols effectiveness and discontinuation

The NRS score variations during the follow-up period are shown in Table 3. The total (mean ± SD) NRS score was 7.6 ± 1.1 at T0, 5.8 ± 1.4 at T1, and 5.5 ± 1.5 at T2 (P = 0,008 by linear test for trend).

**Table 3:** NRS score variations (mean ± SD) during the follow-up.

	Total	No responders	Initial responders	Relevant responders
N patients (%)	290	68 (23.4%)	159 (54.8%)	63 (21.7%)
NRS T0	7.6 ± 1.1	7.5 ± 1.4	7.7 ± 0.8	7.3 ± 1.4
NRS T1	5.8 ± 1.4	7.3 ± 1.4	5.8 ± 0.8	4.2 ± 0.8
NRS T2	5.5 ± 1.5	7 ± 1.5	5.5 ± 1.1	4 ± 1

P = 0.008 (linear by linear test for trend). NRS = Numeric Rating Scale. SD = Standard Deviation.

Out of the study population of 297 patients, 7 (2.3%) were lost at the first follow-up visit (T1), while 290 completed the second follow-up visit (T2). Discontinuation of nabiximols was recorded in 30 (10.3%) patients at T1 (early discontinuers) and in 71 (24.5%) patients at T2 (late discontinuers). Overall, 189 (65.2%) were still on treatment during the follow-up period. The baseline predictor of early discontinuation was a higher NRS score at baseline (HR: 1.32, 95% CI 1.01–1.72; p = 0.04) when contrasted with both continuers and late discontinuers. The main reasons for treatment discontinuation are shown in Table 4. Early discontinuation was mainly driven by lack of effectiveness, whereas late discontinuation had mixed reasons (p = 0.02).

At T1, 63 (21.7%) patients had a CRR, 159 (54.8%) an IR and 68 (23.5%) were NR. The probability of CRR, rather than IR or NR, was higher in patients with lower baseline NRS score (OR: 0.69, 95% CI 0.54–0.90, p = 0.005).

**Table 4:** Reasons for discontinuation of nabiximols.

Reason for discontinuation		Late Discontinuers	Early Discontinuers	Total
Dizziness	Patients (%)	6 (8.5)	1 (2.7)	7 (6.5)
Fatigue	Patients (%)	8 (11.3)	2 (5.4)	10 (9.3)
Neurobehavioral effect	Patients (%)	9 (12.7)	3 (8.1)	12 (11.1)
Lack of efficacy	Patients (%)	32 (45.1)	29 (78.4)	61 (56.5)
Others	Patients (%)	16 (22.5)	2 (5.4)	18 (16.7)
Total	Patients (%)	71 (100)	37 (100)	108 (100)

P = 0.02 by the Chi-squared test

### Physiotherapy

A total of 210 (71%) patients entered specific outpatient or home-based PT programs for spasticity with a weekly frequency of 2.7 ± 0.9 sessions, at least 4 weeks before and during nabiximols treatment. Patients undergoing PT programs were more often SPMS, had a more severe EDSS score and worse NRS score (see Table 5).

**Table 5:** Baseline characteristics in patients undergoing and not undergoing a PT program.

	Physiotherapy		p
	No (n = 87)	Yes (n = 210)	
Sex (M:F)	40:47	81:129	0.24
Disease course (RR:SP:PP)	28:46:13	38:140:32	0.025
Age (mean ± SD)	53.2 ± 9.8	53.5 ± 9.8	0.80
Disease duration (mean ± SD)	19.1 ± 9.2	17.7 ± 8.5	0.17
Baseline EDSS (mean ± SD)	6.0 ± 1.6	6.4 ± 1.1	0.02
Baseline NRS score (mean ± SD)	7.3 ± 1.3	7.7 ± 1.0	0.03

PT = Physiotherapy. M = Male. F = Female. RR = Relapsing Remitting. SP = Secondary Progressive. PP = Primary Progressive. SD = Standard Deviation. EDSS = Expanded Disability Status Scale. NRS = Numeric Rating Scale.

Overall, 42 (61.8%) of non responders, 114 (71.7%) of initial responders and 52 (82.5%) of relevant responders were undergoing PT versus 26 (38.2%) of non responders, 45 (28.3%) of initial responders and 11 (17.5%) of relevant responders who did not undergo a PT (p = 0.03 by the Chi-squared test). Patients undergoing PT programs had a higher probability of CRR but not IR (OR = 2.6 95% CI 1.3–5.6, p = 0.01) after controlling for all the other baseline variables.

**Fig 1:**
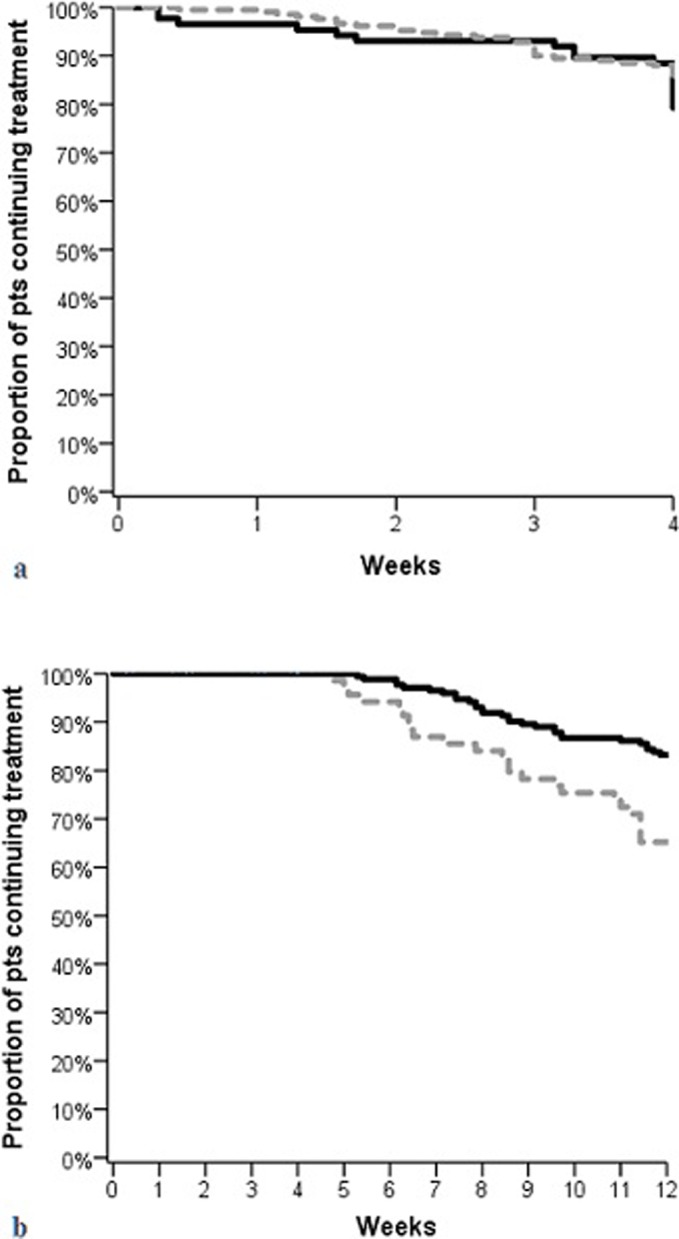
Discontinuation predictors: early discontinuation **(a)**. Late discontinuation **(b)**. Black lines: patients undergoing a physiotherapy; dotted grey lines: patients not undergoing physiotherapy.

The probability of being late discontinuers was lower in patients undergoing combined therapy (nabiximols plus PT) as compared to those treated only with nabiximols (HR: 0.41; 95% CI 0.23–0.69, p = 0.001) (see Fig 1 and Table 6).

**Table 6:** Discontinuation of nabiximols in patients with and without associated PT.

		Physiotherapy at T0		Total
		No	Yes	
Continuers	Patients (%)	45 (51.7)	144 (68.6)	189 (63.6)
Late discontinuers	Patients (%)	24 (27.6)	29 (13.8)	53 (17.8)
Early discontinuers	Patients (%)	18 (20.7)	37 (17.6)	55 (18.5)
Total	Patients (%)	87 (100)	210 (100)	297 (100)

p = 0.008 by the Chi-squared test. PT = Physiotherapy.

## Discussion

This multicenter study confirms the previous evidences of the effectiveness of nabiximols in reducing spasticity in MS patients. We found that 77% of patients reached the IR threshold and 22% the CRR threshold at the end of the 4 weeks trial period. The mean NRS spasticity score reduction was 23.7% at T1 and a 27.6% at T2 (3 months) compared to T0 (baseline). These results were in line with a larger multicenter observational study on the use of nabiximols in Italy [11]. In this latter study, 70.5% of patients with similar baseline characteristic reached IR threshold. Discontinuation of nabiximols during the observation period proved also to be on the same level of the Italian Study (36% versus 39.5%).

The most common adverse events (AEs) were mild to moderate dizziness and fatigue, with overall AEs reported by 47% of patients. Recently, observational studies have shown that patients tend to use lower doses of nabiximols compared with clinical trials, resulting in a lower incidence of AEs, with no evidence of addiction, abuse or misuse [10].

Patients undergoing PT intervention combined with nabiximols had higher probability of having a CRR, compared to those treated only with nabiximols. A recent meta-analysis demonstrated that PT interventions are a safe and beneficial option for spasticity in patients with MS [14]. Due to the multicenter nature of our survey, we considered a heterogeneous array of PT programs including all our patients; accordingly, we preferred to divide bimodally the study population in those attending or not attending PT. PT interventions included exercise programs on self-perceived spasticity and muscle tone respectively in both outpatient and home-based regimens. We have previously demonstrated that 15 PT sessions in addition to botulinum toxin type A injection were more effective than botulinum toxin alone, suggesting a potential synergistic effect [19].

A possible explanation of the synergistic effect of oromucosal cannabis with rehabilitation treatment was suggested by Mori et al. [20]. Exercise increases endocannabinoid signaling in humans [21] and attenuates clinical manifestations of experimental MS [22] through increased *Cannabinoid receptor type 1* (*CB1*) sensitivity [23]. In contrast, genetic deletion of *CB1* receptor is associated with decreased improvement of motor performance induced by exercise [24]. Moreover, the *CB1* receptor seems to be involved in the control of spasticity both in EAE [25] and in patients with MS [11, 24].

The probability of being late discontinuers was reduced in patients undergoing combined therapy compared to those treated with nabiximols alone confirming that a combined treatment may increase effectiveness on MS spasticity. The major cause of late discontinuation in our sample was, indeed, related to the lack of effectiveness. These results are in line with our previous observations showing that the major determinant of botulinum toxin type A discontinuation in clinical practice was the lack of regular PT [26].

The retrospective design of our study implies a number of limitations. The main drawback regards the absence of a standardized and monitored PT program followed by all patients. Each PT program was selected and customized accordingly to the participant's specific needs and physical capabilities but included a minimal set of activities (see Design and Setting). However, the complete list of PT followed by our group of patients cannot be obtained: a prospective assessment aimed to evaluate specific techniques’ single contribution to the PT improvement of spasticity in MS patients treated with anti-spasticity drugs would be required to properly assess this issue.

The advantage of this design is to reflect more closely the real clinical setting of rehabilitation in MS patients, but future randomized, controlled trials are required to explore the impact of pre-defined specific PT programs applied to MS patients treated with Nabiximols.

## Conclusions

Our study provides further insights about effectiveness and tolerability of nabiximols in the treatment of MS-related spasticity. We found that the association of PT intervention was the main predictor of effectiveness and persistence with nabiximols treatment. Despite its novelty, our study suffers from a number of limitations due to its retrospective design, as this may have been a source of selection and recall biases. A prospective, randomized, controlled trial may be required to confirm our data.

## Supporting information

S1 File Supporting information. (XLS)
